# Enhancing epidemiological analysis of intercontinental dispersion of H5N1 viral strains by migratory waterfowl using phylogeography

**DOI:** 10.1186/1753-6561-8-S6-S1

**Published:** 2014-10-13

**Authors:** Dhananjai M Rao

**Affiliations:** 1 CSE Department, Miami University, 510 E. High Street, Oxford 45056 OHIO, USA

**Keywords:** Epidemiology, H5N1, Phylogeography, Temporo-Geospatial Analysis

## Abstract

**Background:**

Intercontinental migratory waterfowl are the primary vectors for dispersion of H5N1 viruses and have been implicated in several zoonotic epidemics and pandemics. Recent investigations have established that with a single mutation, the virus gains the ability to transmit between humans. Consequently, there is a heightened urgency to identify innovative approaches to proactively mitigate emergent epidemics. Accordingly, a novel methodology combining temporo-geospatial epidemiology and phylogeographic analysis of viral strains is proposed to identify critical epicenters and epidemic pathways along with high risk candidate regions for increased surveillance.

**Results:**

Epidemiological analysis was used to identify 91,245 candidate global infection transmission pathways between 22 high risk waterfowl species. Dominant infection pathways (25,625 and 54,500 in summering and wintering zones) were identified through annotation using phylogeographical data computed from the phylogram of 2417 H5N1 HA isolates (from GISAID EpiFlu database). Annotation of infection pathways in turn delineated 23 influential clades out of 130 clades in the phylogram.

**Conclusions:**

The phylogeographic analyses provides strong cross-validation of epidemic pathways and identifies the dominant pathways for use in other epidemiological and prophylactic studies. The temporo-geospatial characteristics of infection transmission provides corroborating, but novel evidence for rapid genesis of H5N1 lineages in S.E. Asia. The proposed method pinpoints several regions, particularly in the southern hemisphere, as candidates for increased surveillance.

## Background

Humanity continues to face a multitude of global socioeconomic challenges due to annual epidemics and punctuated pandemics of highly virulent zoonoses such as avian influenza (H5N1, H7N9) and the 2009 swine flu (H1N1) pandemic [1,2]. The 2009 swine flu (H1N1) pandemic virus involved segments from avian serotype [1]. Highly Pathogenic Avian Influenza (HPAI) virus are routinely transmitted to humans in several parts of the world as reported by the World Health Organization (WHO) [3] (see figure in supplementary material), including a recent case in Canada [4], with an alarming 60% mortality rate [5]. Moreover, the disease is of global importance because low pathogenic forms of the viruses cause billions of dollars of annual losses due to recurrent epidemics in poultry [5,6].

### Ecology of avian influenza: need for analysis of migratory waterfowl

**Figure 1:**
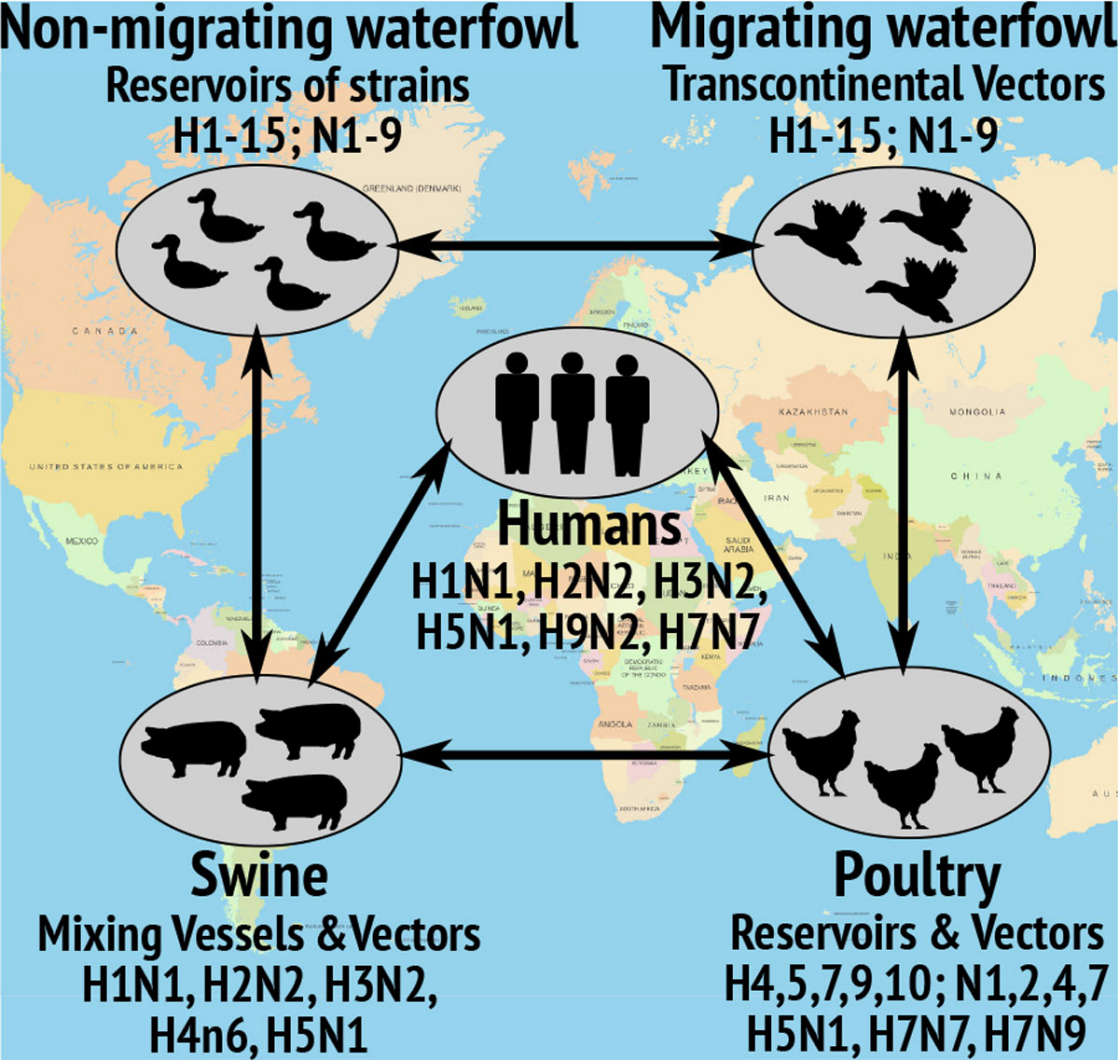
**Overview of global ecology of Avian Influenza Viral serotypes, hosts, and vectors are shown**.

The global ecology of avian influenza, is summarized in Figure 1 along with various polymorphic strains. It has been established that migratory waterfowl, especially anseriformes and charadrillformes, play a central role in the global ecology [7,8]. Moreover, migratory waterfowl have been implicated as natural reservoirs, mixing vessels, and intercontinental vectors for various serotypes of avian viruses [8]. Unfortunately, knowledge on global spread of H5N1 is rather limited [9] with ongoing debates regarding its transmission pathways [8,10].

Although past outbreaks have been sporadic and unsustained, several recent investigations have established that with just one mutation the H5N1 virus gains the ability to be readily transmitted between humans and a global pandemic is imminent [11]. Consequently, there is heightened urgency to shift the focus of investigations from studying possibilities to analyzing probabilities of outbreaks to proactively mitigate or even preempt emergent pandemics [12] in contrast to delayed responses to the 2009 H1N1 pandemic as discussed in the report by the executive office of the president of the United States [13]. Accordingly, several multinational surveillance efforts have been initiated to collate data on various characteristics of migratory species [8]. The surveillance efforts include: large scale satellite tracking, banding and tracking of individual birds in conjunction with satellite telemetry, and various biological sampling and cataloging efforts [8].

### SEARUMS: ecological and epidemiological modeling and analysis system

Despite the advancements in technologies and improvements in economies of scale, data from surveillance is relatively coarse and sparse. Furthermore, field observations and satellite telemetry only provide a snapshot of various natural processes that influence global ecology of the disease. Furthermore, comprehensive epidemiological analysis using effective computational models play a pivotal role in design and implementation of national and multinational prophylactic strategies and policies. Consequently, the surveillance data needs to be combined with computational analysis methods to generate comprehensive, multifaceted information and draw actionable inferences. However, a versatile and comprehensive software system is required to enable and the aforementioned computational analyses.

Accordingly, a ecological and epidemiological analysis environment called SEARUMS [14] (http://www.searums.org/) has been developed and is used in this investigation. The biomathematical models for temporo-geospatial epidemiological analysis supplied to SEARUMS are called Eco-descriptions. The software pipeline includes modules for generating Eco-descriptions from Geographic Information Systems (GIS) datasets that has been used in this study (refer to Methods section for details). SEARUMS uses an agent-based descriptive behavioral computational-modeling approach [15] to elicit epidemiological characteristics [14]. The agents in SEARUMS implement the classical bio-mathematical compartmental models that are widely used in epidemiology [16]. In a compartmentalized model the population being analyzed is partitioned into non-intersecting subsets called compartments. Compartments are defined such that the sub-population within a compartment exhibits a vital disease characteristic, such as: Susceptible (S), Exposed (E), Infected (I), and Recovered (R). The epidemiological characteristics of the classical SEIR model is modeled using the following system of differential equations:

$$\begin{array}{c} dS/dt=\mu N-\left[\lambda +\mu \right]S\left(t\right)\;\;\;\;dI/dt=\beta E\left(t\right)-\left(v+\mu \right)I\left(t\right)\\ dE/dt=\lambda S\left(t\right)-\left(\beta +\mu \right)E\left(t\right)\;\;dR/dt=vI\left(t\right)-\mu R\left(t\right) \end{array}$$

The constants *µ, λ, β*, and v represent the birth/death rate, the force of infection, latency period, and recover rates respectively. These constants are determined based on the characteristics of the disease being modeled and are supplied via the Ecodescription. In SEARUMS spread of infection to various agents occurs when agents overlap with each other. The system is modeled as discrete time Markov processes driven by an underlying multi-threaded Discrete Event Simulation (DES) kernel. Various phenomena that occur during simulation are logged for further analysis. A more detailed description of SEARUMS is available in the literature [14].

## Methods

**Figure 2:**
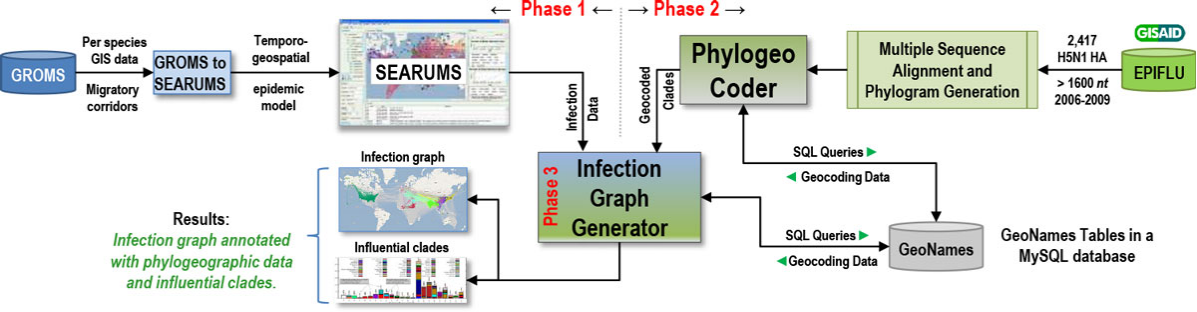
**Overview of the three phases in the method used in this study**.

The focus of this study is to utilize phylogeographic analysis of H5N1 viral strains to validate and enhance information about epidemic transmission pathways identified using temporo-geospatial epidemiological analysis of high risk migratory waterfowl. The validated and enhanced knowledge is then used to draw further conclusions in addition to serving as a framework for various ecological, phylodynamic, and prophylactic analyses. Figure 2 presents an overview of the various steps involved in the proposed methodology that are broadly classified into three phases. The first phase involves identification of candidate infection pathways via epidemiological analysis of migratory patterns of high risk waterfowl species. The second phase involves phylogeospatial analysis of viral strains to geocode strains in clades. The third phase combines the results from the first two phases to validate and enhance infection pathways and conduct various analyses. These three phases are discussed in the following subsections.

### Phase 1: identification of infection pathways in high risk migratory waterfowl

**Figure 3:**
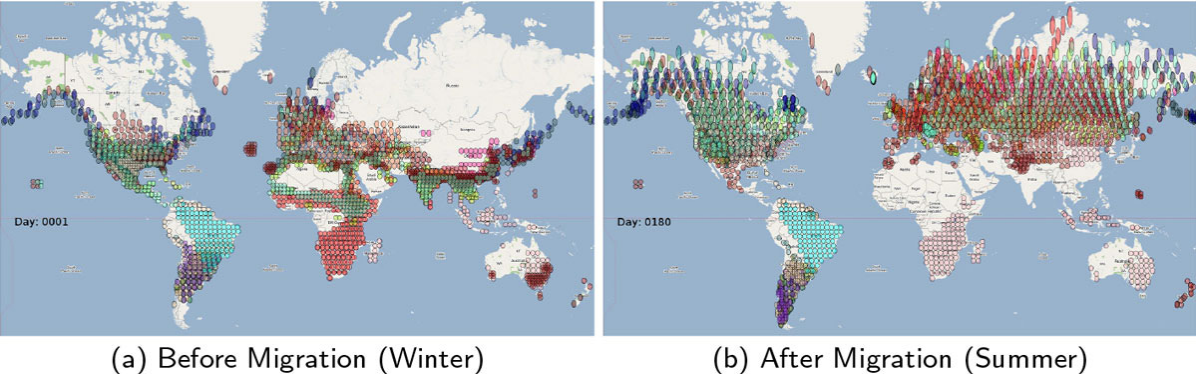
**Locations where flocks (various species color coded) remain for significant portion of time**. Subfigure (a) shows flocks in their initial wintering zone. Subfigure (b) shows flocks in the summering zones at the end of one seasonal migration. Model details and videos available at http://www.searums.org/glbio14/.

The epidemic model used in this study consisted of all 22 high risk waterfowl species shown in Table 1 that have been collated from earlier publications [17-20]. The GIS data for the 22 species were obtained from GROMS database [21] while migratory characteristics were obtained from BirdLife International Database (BID) [22]. The epidemic model represented as an Eco-description was generated from the GIS datasets using SEARUM's model generation module. The position of various flocks in the wintering zones and after migration to summering zones are shown in Figure 3(a) and Figure 3(b). Additional model images are included in supplementary material with further information available at http://www.searums.org/glbio14/, including complete model, high resolution images, and video illustrating the migration and infection spreads discussed in this paper.

**Table 1:** List of high risk waterfowl species used for analysis.

Species Name	Population	#Flocks	Species Name	Population	#Flocks
Aix sponsa	3500000	64	Anas bahamensis	640000	78

Anas acuta	5300000	372	Amazonetta brasiliensis	110000	103

Anas platalea	500000	47	Anas platyrhynchos	19000000	557

Anas sibilatrix	250000	30	Anas versicolor	126000	42

Anser anser	1000	4	Callonetta leucophrys	50000	3

Aythya ferina	2200000	213	Aythya fuligula	2600000	148

Aythya marila	1200000	114	Branta canadensis	5500000	169

Anser indicus	56000	11	Cygnus melanocoryphus	50000	32

Melanitta nigra	2100000	96	Mergellus albellus	130000	71

Netta peposaca	1000000	26	Philomachus pugnax	4200000	210

Anas Crecca	5900000	403	Porzana pusilla	21300	262

The list of species was obtained from earlier publications [17-20]

The epidemiological analysis was conducted by seeding an infection in one Anas platyrhynchos flock in Guangdong, China (at 23°21'36.53"N, 113°36'25.89"E), corresponding to the root (A/goose/Guangdong/1996) of the revised WHO H5N1 nomenclature phylogram [23]. The model was configured to have a basic reproductive number for the infection (R_0_) to be greater than 1 to reflect the enzootic nature of the infection. Furthermore, the disease transmission parameters were configured to reflect a Susceptible→Infected (SI) type compartmental epidemiological model. The model was simulated for a period of three years while logging the locations of various infections occurring in the model. The stochastic nature of the simulations require the use of a Monte Carlo approach in which infections consistently occurring in multiple simulations are identified as the dominant set of infections. Temporogeospatial attributes of flocks involved in each pair of infection transmission are collated to yield an infection graph for further processing in phase 3. An infection graph (see Figure 7) is a Directed Acyclic Graph (DAG) in which location of flocks are nodes with edges connecting pairs of flocks involved in infection transmission.

### Phase 2: phylogeographical analysis of H5N1 strains

The phylogram generation procedure adopted by WHO/OIE/FAO H5N1 Evolution Working Group [23] has been utilized to generate a phylogenetic tree using 2,417 H5N1-Hemmagglutinin (HA) segments. The viral strains were obtained from GISAID EpiFlu database [24] by restricting the search to reads longer than 1600 nucleotides (*nt*), which corresponds to 90% of the open frame read length [23]. Furthermore, the EpiFlu search query was restricted to a 3 year time period from 2006 to 2009 (inclusive) corresponding to the 3 year period used for epidemiological analysis in Phase 1. A multiple sequence alignment of the 2,417 H5N1-HA sequences was generated using MUSCLE [25] (version 3.7) using 16 iterations. A large unrooted neighbor-joining tree of the 2,417 H5N1 HA strains was constructed using a GTR+I+Γ model in PAUP* v4.0b10 [26]. The newick form of the phylogram generated by PAUP* was used to categorize leaves into clades such that percentage pairwise nucleotide distances between and within clades are > 1.5% and < 1.5% respectively, concordant with WHO/OIE/FAO clade definition criteria [23].

The clades identified from the phylogram are marked with unique numbers and the isolate names are used to geocode each leaf. Geocoding was conducted using the GeoNames dataset [27] stored in a local MySQL database with suitable geospatial indexes to accelerate various SQL queries. The geocoding process was conducted in three passes to obtain both region and country encoding to provide higher geospatial resolution for each sequence. For example, the isolate name A/chicken/Tabanan/BBVD-142/2007 was geocoded to Indonesia, Taban (rather than just Indonesia). Similarly, A/Canada goose/AK/44075-058/2006 was geocoded to United States, Alaska. In order to obtain regional-level geocoding, the first pass attempted to perform exact matches on various GeoNames tables using each term in the isolate name. However, if the first pass did not yield an exact match a second pass is performed using a set of manually supplied overrides that were necessary to disambiguate entries. For example, A/ruddy turnstone/DE/509531/2007 must be geocoded to United States, Delaware rather than Germany (country code DE). If the second pass is unsuccessful then a third pass with approximate matching is pursued. Manual disambiguation is solicited in the third pass if the approximate queries result in multiple matches. The geocoding information for each read along with its clade numbers are persisted for use in the next phase of processing.

### Phase 3: fusing phylogeographic clades and epidemiological infection graph

The last phase of the proposed methodology utilizes the phylogeographic data extracted in Phase 2 to identify and annotate strong infection pathways in the infection graph generated in the Phase 1. In this phase, the latitude and longitude values for each pair of verities constituting an edge in the infection graph are reverse geocoded to identify correspondence with phylogeographic data. Geocoding is performed using the same GeoNames database in multiple passes using increasing radius (1/50, 1/25, and 1 mile) of matching to identify higher resolution regional-level geocodes. Vertexes that are on ocean surfaces cannot be reverse geocoded and are excluded from further analysis in this phase.

Pairs of reverse geocoded vertexes are matched with clades containing same pairs of geocoded regions in the phylogeographic data. Identification of corresponding clades, called *influential clades *(see Figure 6) is performed by first matching on both region and country (*i.e.*, higher resolution data match) and then just on the country to ensure strong correspondence. For example, the infection pathway between 〈30°52'12"N, 28°22'14.5194"〉 - 〈36°41'4.9194"N, 36°41'4.9194"〉

is geocoded to Matruh, Egypt and Mugla, Turkey respectively with correspondence to a clade containing H5N1 HA sequences from Egypt and Turkey such as: A/duck/Egypt/08355S-NLQP/2008 and A/chicken/Turkey/Ipsala563/2008 to enumerate a few. The infection pathway is annotated and persisted for visualization and various analyses discussed in the next section.

## Results

**Figure 4:**
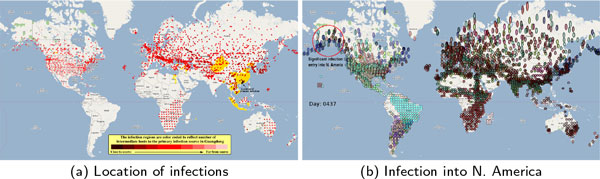
**Overview of infection spread from Guangdong, China**. Subfigure (a) shows distance of infection from primary source in terms of number of intermediate hosts (indicative of increase in viral diversity and lineage) and areas with human outbreaks (in bright orange) as reported by WHO [3]. Subfigure (b) shows initial entry of infection into North America migration.

The first phase of epidemiological analysis involving semiregular tessellation of GIS data resulted in generation of 3055 entities representing flocks of 22 high risk waterfowl species tabulated in Table 1. Several calibration runs were conducted to tune epidemiological parameters to reflect realistic inter and intra-flock infection spread. Each epidemiological analysis cycle required about 4.62 hours to complete on a 3.9 GHz 8 core processor using 24 concurrent threads. The location of dominant infection transmissions between flocks are plotted in Figure 4(a). The infection locations in the figure are color coded to reflect the number of intermediate hosts to the source infection in Guangdong, China (at 23°21'36.53"N, 113°36'25.89"E), corresponding to the root (A/goose/Guangdong/1996) of the revised H5N1 nomenclature phylogram [23]. The locations of various infection transmissions indicate potential areas for secondary outbreaks and increased density of outbreaks in turn increase the probability of human outbreaks [10]. The high risk areas as reported by WHO [3] are highlighted in bright orange in Figure 4(a). The figure highlights the overlap between dense outbreaks identified by epidemiological analysis and the regions with observed human cases reported by WHO [3].

The infection patterns radiate from the primary infection source through S. E. Asia, Eastern Europe, Western Europe and into North America (as summarized in Figure 4) confirm that the infections into North America are significantly distant from S. E. Asia. Furthermore, temporal characteristics of the infections indicate that initial infections into North America has a lag of ~15 months (see video referenced in supplementary material) with infections seeding occurring via both transatlantic and transpacific migratory corridors. The primary entry locations were observed to be near the Gulf of Alaska, a known high risk area [18], which consistently shows sufficiently strong infections. Observations of ~15 month lag between S. E. Asia and primary entry pathways into North America are also corroborated by surveillance [8], statistical, and bioinformatics analyses reported by other researchers [6,18,28]. In the epidemic analysis logically spanning 3 years, infection spread from North America was constrained to northern South America, with potential for larger infection spread into South America.

**Figure 5:**
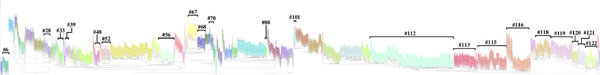
**Overview of 130 clades in phylogenetic tree of 2,417 H5N1-HA sequences from GISAID EpiFlu database**. The phylogram was created via neighbor-joining and GTR+I+Γ model using PAUP*. The clades are color coded and annotated to highlight influential clades that contributed to phylogeocoding of infection pathways. A zoomable image of the complete phylogenetic tree is available in supplementary materials. Additional information about the clades are also available in Figure 6.

The multiple sequence alignment of 2,417 H5N1-HA sequences (average length: 1710.28 ± 34.37 *nt*, minimum length: 1602 *nt*, maximum length: 1809 *nt*), with minimum length of 1600 *nt *(>90% open frame read length), spanning a three year period corresponding to the epidemiological analysis interval, and obtained from GISAID EpiFlu database [24] resulted in an alignment spanning 1841 nucleotides in 5 refinement iterations out of the maximum configured 16 iterations. The phylogram generated from the alignment using neighbor joining and the GTR+I+Γ model in PAUP* was categorized into 130 clades using the criteria proposed by WHO/OIE/FAO [23] which requires that percentage pairwise nucleotide distances between and within clades are > 1.5% and < 1.5% respectively. An overview of the resulting phylogram is shown in Figure 5. The resulting phylogram was manually cross-verified to be consistent with the WHO/OIE/FAO reference taxonomy phylogenetic tree [23].

**Figure 6:**
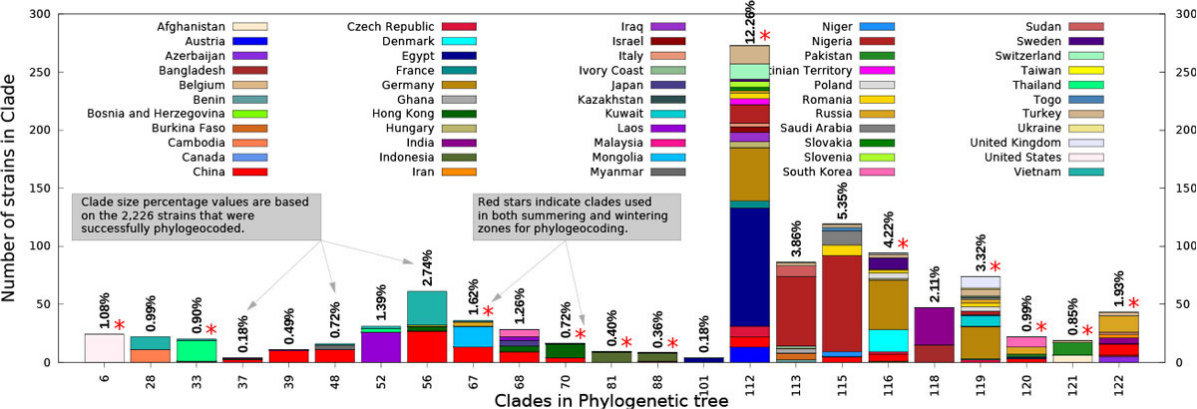
**Overview of the 23 influential clades that were used to annotate the edges in the infection graphs shown in Figure 7**. Full details on all the clades and a complete phylogenetic tree are available in supplementary materials.

**Figure 7:**
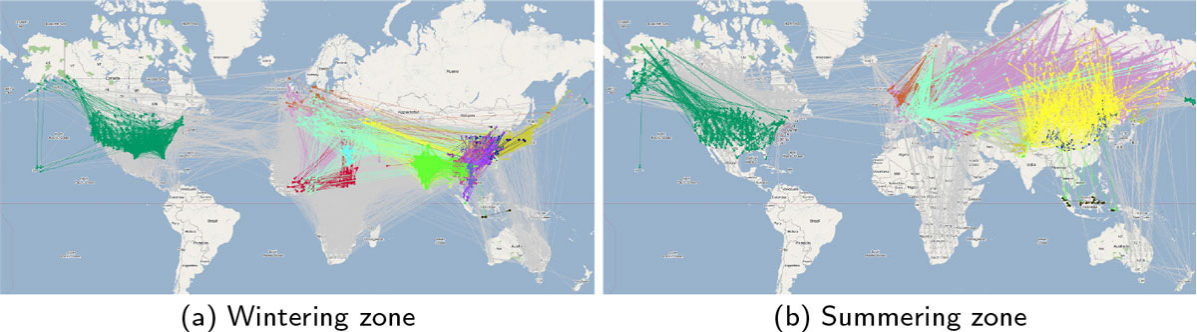
**Infection graphs with phylogeographic annotations color coded to match influential clades**. Grey edges do not have corresponding phylogeographic annotations (primarily due to lack of viral isolates from southern hemisphere). Subfigure (a) shows flocks in their initial wintering zone. Subfigure (b) shows flocks in the summering zones at the end of one seasonal migration.

The leaves in the phylogram shown in Figure 5 were geocoded using the read names to obtain region and countries. Out of the 2,417 H5N1-HA strains 2,226 (92%) reads were successfully geocoded while the remainder could not be geocoded either due to lack of information or ambiguity in the reads. Given the high yield, manual geocoding of the remaining 191 strains was not pursued. The geographic distribution of the strains in the influential clades are summarized by the chart in Figure 6 (details on all the 130 clades is included in supplementary materials). Out of the 130 clades 43 clades were singletons while 15 clades had only two leaves in them and these clades were excluded from further analysis. The remaining clades had an average size of 32.4 ± 43.15 leaves with a median size of 18.5 with majority of the clades spanning multiple countries. Clade #122 (corresponding to clade 2.2 of WHO/OIE/FAO taxonomy) was the largest and most diverse clade consistent with the revised H5N1 taxonomy phylogram with almost 50% of the reads from Egypt, consistent with increased surveillance efforts between 2006 to 2008.

The 72 geocoded clades were used for phylogeographic annotation of the infection graph generated from epidemiological analysis of the infection spread. The infection graph corresponding to summering and wintering zones generated by annotating edges (infection pathways) using phylogeographic annotations is shown in Figure 7. Note that these are the regions were the flocks predominantly roost and are primary locations for cross species infections. Out of the 72 geocoded clades only 28 were influential in annotation in wintering zone (Figure 7(a)) and 12 were influential in summering zone (Figure 7(b)). Furthermore, the 13 influential clades (denoted by red stars in Figure 6) overlapped with the 23 influential clades from the summering zone. Moreover, the largest and most diverse clade #112 did not dominate annotation in either zones and thereby minimizing concerns of skews due to sampling bias in the phylogeographic data. Interestingly, the 23 influential clades yielded annotations for just 27.8% of the edges in the wintering zone in contrast to 12 influential clades managing to annotate 59.73% of the edges in summering zone.

## Discussions and conclusions

The aggregate data obtained from the result of fusing phylogeographic and epidemiological data are shown in Table 2. The dominant country from the various clades is China, spanning 13 of the 23 influential clades followed by Vietnam in 4 clades. The data indicates that these countries have large diversity in the enzootic strains. Furthermore, the corresponding set of edges in the infection graphs shown in Figure 7(a) are predominantly located in S. E. Asia with bidirectional edges indicating cyclical infection patterns. Fusing phylogeographic data and the infection pathways from epidemiological analysis provides novel evidence indicating the increased potential for reassortments to occur in this region.

**Table 2:** Details on 23 influential clades involved in phylogenetic coding of pathways in the infection graph.

Clade Num.	Strain Count	Countries in the clade (# strains)	# Edges Annotated	
			**Wintering**	**Summering**

6	24 (1.08%)	United States (24)	6190 (24.38%)	3700 (6.79%)

28	22 (0.99%)	Cambodia (11), Vietnam (11)	210 (0.83%)	0 (0.00%)

33	20 (0.90%)	India (1), Thailand (18), Vietnam (1)	3640 (14.34%)	5 (0.01%)

37	4 (0.18%)	China (2), Hong Kong (1), Malaysia (1)	120 (0.47%)	0 (0.00%)

39	11 (0.49%)	China (10), Laos (1)	350 (1.38%)	0 (0.00%)

48	16 (0.72%)	China (11), Myanmar (4), Vietnam (1)	1030 (4.06%)	0 (0.00%)

52	31 (1.39%)	Laos (26), Thailand (3), Vietnam (2)	460 (1.81%)	0 (0.00%)

56	61 (2.74%)	China (27), Hong Kong (4), Taiwan (1), Vietnam (29)	40 (0.16%)	0 (0.00%)

67	36 (1.62%)	China (13), Mongolia (18), Russia (4), Vietnam (1)	585 (2.30%)	3890 (7.14%)

68	28 (1.26%)	China (9), Hong Kong (5), Japan (5), Laos (3), South Korea (6)	35 (0.14%)	0(0.00%)

70	16 (0.72%)	China (4), Hong Kong (12)	1710 (6.74%)	705 (1.29%)

81	9 (0.40%)	Indonesia (9)	10 (0.04%)	80 (0.15%)

88	8 (0.36%)	China (1), Indonesia (7)	15 (0.06%)	50 (0.09%)

101	4 (0.18%)	Egypt (4)	90 (0.35%)	0 (0.00%)

112	274 (12.31%)	Austria (13), China (9), Czech Republic (9), Egypt (102), France (6), Germany (46), Hungary (5), Iraq (8), Israel (5), Italy (3), Nigeria (16), Palestinian Territory (5), Romania (5), Russia (2), Slovakia (3), Slovenia (5), Sweden (2), Switzerland (13), Turkey (17)	2275 (8.96%)	4655 (8.54%)

113	86 (3.86%)	Benin (2), Burkina Faso (6), Ghana (4), Ivory Coast (2), Nigeria (60), Sudan (9), Turkey (3) China (5), Niger (87), Nigeria (83), Romania	770 (3.03%)	0 (0.00%)

115	202 (9.07%)	(9), Saudi Arabia (12), Togo (3), Turkey (3) Bosnia and Herzegovina (1), China (6), Czech Republic (2), Denmark (19), Germany (43),	5 (0.02%)	0 (0.00%)

116	94 (4.22%)	Hungary (1), Poland (5), Romania (2), Russia (1), Sweden (10), Turkey (3), United Kingdom (1)	345 (1.36%)	1425 (2.61%)

118	47 (2.11%)	Bangladesh (15), India (32)	455 (1.79%)	0 (0.00%)

119	74 (3.32%)	Czech Republic (2), France (1), Germany (28), Kuwait (9), Mongolia (1), Nigeria (3), Poland (4), Romania (3), Russia (3), Saudi Arabia (2), Switzerland (1), Turkey (6), Ukraine (1), United Kingdom (10) China (3), Japan (1), Mongolia (1), Pakistan	1695 (6.68%)	38555(70.74%)

120	22(0.99%)	(2), Russia (6), South Korea (9)	2190 (8.63%)	295 (0.54%)

121	20 (0.90%)	Afghanistan (6), Pakistan (12), Turkey (2)	80 (0.32%)	105 (0.19%)

122	43 (1.93%)	Azerbaijan (5), Bangladesh (1), China (10), India (5), Iran (3), Italy (2), Russia (14), Turkey (3)	3085 (12.15%)	1035 (1.90%)

The strain count percentages are based on 2,226 strains (out of 2,417) that were successful geocoded. The percentages of edges annoted is based on 25,625 and 54,500 (out of 91,245) edges in summering and wintering zones respectively that were successfully phylogeocoded.

The data in Table 2 shows a significant variation in the number of edges annotated by influential clades between summering and wintering seasons. The number of influential clades increases in wintering zone because the birds migrate down south and spread out across many countries. This time frame also coincides with increase in influenza epidemics in the temperate and subtropical regions thereby increasing the potential for reassortment between human and avian influenza viruses leading to emergent of novel and possibly highly virulent strains that cause mortality in humans. These inferences are consistent with prior investigations reported by various researchers and multinational surveillance organizations [2,9,10,18-20]. However, the significant evidence correlating migratory patterns of high risk waterfowl species to the "breeding grounds" of novel H5N1 strains using phylogeographic-epidemiological analysis is an original and unique inference from this research. in addition to providing a complementary perspective on the ecological aspects of avian influenza, the inferences increase confidence in the proposed methodology.

The locations of infections that occur during migration, which correspond to migratory stopover sites, are highlighted in Figure 4(a) show strong correspondence with the high risk regions reported by WHO [3]. Many of the infection pathways that arise due to intercontinental waterfowl migration show strong relationship between Asia and Europe. Successful phylogeographic annotation of these pathways substantiate their validity because isolation of many closely related (less than 1.5% *nt, i.e.*, fewer than 28 bases out of 1841 *nt*) H5N1 strains and the exploratory data analysis provide statistically significant evidence to clearly reject the null hypothesis that the infection pathways are a mere coincidence.

The phylogeographically annotated epidemiological data related to entry of infections into Americas is also consistent with surveillance [8], statistical, and bioinformatics analyses reported by other researchers [6,18,28]. Specifically, the infection graph correctly elicits Alaska as a primary gateway point into Americas [18,19] and the temporal characteristics show reported minimum of ~15 month lag between S. E. Asia and Americas [28]. The bidirectional infection patterns between North and South Americas suggest continuous circulation of viruses providing support for the isolated clade #6. These aforementioned observations add further credence in the proposed methodology and validity of the underlying epidemiological model.

The phylogeographic dataset used in this study did not contain viral isolates to support the transatlantic infection pathways shown in Figure 7. Analysis of the long read (at least 1600 *nt*) H5N1-HA strains isolated from United States since 2006 in the GISAID EpiFlu database indicates that they are closely related to the strains in clade #6 shown in the first row of Table 2. However, the number of samples in the database from West European and Scandinavian countries is very few and that impacts the extent of annotation of transatlantic pathways. However, given the foregoing analysis that provides strong support for the underlying epidemiological model, these infection pathways cannot be dismissed and consequently emphasize the need for concentrated surveillance efforts in these regions.

The GISAID EpiFlu database does not contain sufficient number of viral strains from the southern hemisphere in the analysis timeframe spanning 2006 to 2009. The database contains (completely ignoring date ranges) just two strains from the whole of South America, 34 strains from Africa (excluding countries already included in this study), one from Oceania (includes: Australia, New Zealand and several smaller island nations), and zero from Antarctica. Consequently, a large fraction of the infection pathways to countries in the southern hemisphere could not be phylogeographically annotated. The lack of phylogenetic data from the southern hemisphere is more pronounced during wintering seasons as highlighted in Table 2 because birds migrate towards the equator. The lack of strains motivates increase surveillance when viewed in the context of the aforementioned analyses that provide strong evidence for the proposed methodology in the northern hemisphere. Moreover, the nodes in the infection graph with large degrees of unannotated edges serve as landmarks for guiding surveillance efforts.

Inferences drawn from this research provide strong validation for the proposed methodology and the underlying epidemiological model. The study indicates that the fusion of phylogeography with epidemiology can provide novel, yet intuitive results and is a distinctive approach for ecological and epidemiological analysis. For instance, nodes with high degrees serve as epicenters for enacting various prophylactic and containment strategies. Moreover, having identified novel strains in S.E. Asia, nodes along the transmission pathways in the infection graph can be actively monitored to strategically assess propagation characteristics thereby enabling proactive design of vaccines and prophylactic measures to contain epidemics in humans and livestock. The proposed methodology involving an unique combination of temporo-geospatial epidemiology and phylogeography provides support for pioneering new in *silico *approaches for study and analysis of disease ecology, epidemiology, viral phylodynamics, and prophylaxis.

## Competing interests

The authors declare that they have no competing interests.

## Authors' contributions

Rao conceived the proposed method, conducted experiments, and authored the paper.

## Declarations

This work was funded in part by the Ohio Supercomputer Center (PMIU0110-2) and the Computer Science and Software Engineering (CSE) Department, Miami University, Oxford, OHIO, USA.

This article has been published as part of *BMC Proceedings *Volume 8 Supplement 6, 2014: Proceedings of the Great Lakes Bioinformatics Conference 2014. The full contents of the supplement are available online at http://www.biomedcentral.com/bmcproc/supplements/8/S6.
